# Self-directed learning ability and online student engagement among nursing students: the mediating role of information literacy and the moderating role of self-control

**DOI:** 10.3389/fpsyg.2026.1859825

**Published:** 2026-06-18

**Authors:** Haihong Zhang, Guoshan Gao, Furong Gou, Wenjie Zhang, Xin Tian, Wenxue Zhang

**Affiliations:** 1 School of Nursing, Ningxia Medical University, Yinchuan, Ningxia, China; 2 Shanxi Bethune Hospital, Shanxi Academy of Medical Sciences, Third Hospital of Shanxi Medical University, Tongji Shanxi Hospital, Taiyuan, China; 3 School of Medical Information and Engineering, Ningxia Medical University, Yinchuan, Ningxia, China

**Keywords:** information literacy, nursing students, online student engagement, self-control, self-directed learning ability

## Abstract

**Background:**

Online learning has become an important teaching approach in nursing education, and insufficient online student engagement among nursing students has become a key issue affecting teaching effectiveness. However, the underlying mechanisms related to individual learning characteristics of nursing students have not been fully explored.

**Purpose:**

This study aims to examine the relationship between self-directed learning ability and online student engagement among nursing students, focusing on the mediating role of information literacy and the moderating role of self-control.

**Patients and methods:**

This study recruited 917 undergraduate nursing students from five provinces in China (83% female). Participants completed the self-directed learning ability scale (SDLRS), the information literacy scale (ILS), the brief self-control scale (BSCS), and the online student engagement (OSE) scale, all of which are validated self-report instruments. Data were analyzed using SPSS 26.0 and the PROCESS macro.

**Results:**

Self-directed learning ability had a significant positive direct effect on online student engagement (β = 0.511, *t* = 17.586, *p* < 0.001). Information literacy mediated the relationship between self-directed learning ability and online student engagement, with an indirect effect of 0.346, accounting for 67.7% of the total effect. Self-control negatively moderated the direct effect of self-directed learning ability on online student engagement (β = −0.310, *t* = −2.140, *p* < 0.01).

**Conclusion:**

The results support a moderated mediation model. Self-directed learning ability promotes online student engagement by improving information literacy, and self-control negatively moderates the relationship between self-directed learning ability and online student engagement, such that this effect is stronger at lower levels of self-control. These findings student engagement theory and conservation of resources theory, explain how individual abilities influence learning engagement through cognitive resources in digital learning contexts, and provide empirical support for educational interventions targeting information literacy and self-control.

## 1 Introduction

Online education has become an important teaching form in higher education. It overcomes time and space constraints and is widely used in nursing courses, making online learning a common approach for nursing students (Fernández-Batanero et al., 2022; Metin Karaaslan et al., 2022). Student engagement, defined as the psychological effort students invest in learning, understanding, or mastering academic knowledge and skills, is widely regarded as a core factor for successful online learning (Adesola et al., 2025). From 2016 to 2020, nearly 50% of nursing students in the United States completed part of their studies online, and in 2020, 93% of nursing courses offered online learning (Kerr et al., 2020). Nursing schools in China have also widely implemented online education (Shen et al., 2024). However, insufficient engagement and differences in learning outcomes remain common challenges (Pullan et al., 2023). Studies indicate that active online student engagement depends not only on understanding course content, but also on timely feedback, collaboration, and interaction, which promote knowledge acquisition, skill development, and long-term academic success (Chambers and Whitfield, 2025). However, as research progresses, scholars have increasingly recognized that individual learner characteristics also play a pivotal role in online student engagement (Stan et al., 2022; Pan, 2022). From the perspective of self-regulated learning theory, learners rely on cognitive processing to understand and integrate information (Dunk and Craft, 2025). They also depend on volitional control to maintain goal-directed behavior and resist distractions. These two processes work together to initiate, sustain, and regulate learning activities. As a result, they influence online student engagement. However, how cognitive processing and volitional control interact to shape online student engagement remains unclear, especially among nursing students.

### 1.1 Self-directed learning ability and online student engagement among nursing students

Among many potential factors, self-directed learning ability is considered a key component of online student engagement (Elshami et al., 2022). With the rapid development of medical science and the increasing emphasis on lifelong learning, this ability has become an essential requirement in nursing education (Wong et al., 2021). Self-directed learning ability refers to the capacity to plan, manage, and regulate one's own learning process, including setting goals, selecting strategies, and monitoring progress, which helps students engage more effectively with learning tasks (Zhen, 2025; Cadorin et al., 2017). Students with stronger self-directed learning ability are better at organizing tasks, actively using learning resources, and maintaining continuous involvement, which is reflected in higher online student engagement (Binks et al., 2021). From a theoretical perspective, Student Engagement Theory proposed by Schaufeli suggests that learning engagement is characterized by vigor, dedication, and absorption in learning activities (Schaufeli et al., 2002; Wong and Liem, 2022). The theory emphasizes that learners' internal learning characteristics are important determinants of behavioral engagement. Students with stronger learning initiative are more likely to actively participate in learning activities and maintain higher levels of engagement. In online learning environments, the lack of direct supervision places greater demands on students' self-management and learning initiative (Huang and Wang, 2023). In the present study, self-directed learning ability reflects students' capacity to actively plan, regulate, and manage their own learning process. Nursing students with stronger self-directed learning ability may be more likely to proactively organize online learning tasks, actively participate in online interactions and discussions, and maintain continuous involvement in learning activities, which may contribute to higher online student engagement. In a systematic review, self-directed learning ability was also confirmed from the learners' perspective as an important factor influencing online student engagement (Hu and Xiao, 2025). Based on the above theoretical analysis and empirical evidence, previous studies have shown a significant positive relationship between self-directed learning ability and online student engagement. However, this relationship still needs to be verified in nursing students.

### 1.2 The mediating pathway of information literacy

Information literacy (IL) refers to the ability to identify information needs and effectively access, evaluate, integrate, and apply information in specific learning contexts (Zhao, 2024). In contemporary nursing education, where students are continuously exposed to large volumes of digital and clinical information, IL has become a fundamental competency for both academic learning and professional development (Bergren and Maughan, 2020). Cheek and Doskatsch emphasized that nurses require strong information literacy skills to function effectively in information-rich healthcare environments characterized by rapid knowledge expansion and information overload (Alan Barnard, 1998). Accordingly, IL is increasingly recognized not merely as a technical learning skill, but as an important cognitive and self-regulatory resource that supports adaptive learning behaviors in complex information environments (Rogers and Fyvie, 2024). From the perspective of self-regulated learning theory, effective learning requires learners to actively seek information, critically evaluate its quality, monitor learning progress, and continuously adjust learning strategies according to task demands (Song et al., 2025). These processes rely heavily on information literacy competencies. Students with insufficient IL may experience difficulties in locating credible resources, filtering redundant information, and integrating fragmented knowledge, which can hinder autonomous learning processes and reduce learning efficiency (Kahili-Heede et al., 2022). Previous studies have consistently shown that learners with stronger self-directed learning ability tend to demonstrate higher levels of information acquisition, evaluation, and utilization skills, suggesting a close association between self-directed learning ability and IL (Song et al., 2025). In this context, IL may function as an important psychological and behavioral mechanism linking self-directed learning ability to positive learning outcomes. The importance of IL may be particularly pronounced in online learning environments. Compared with traditional classroom settings, online learning environments are characterized by greater openness, information redundancy, and reduced external supervision (Li et al., 2023). Consequently, students are required to independently manage learning tasks, evaluate information quality, and maintain active participation in learning activities. Existing evidence suggests that students with higher levels of IL are more capable of effectively navigating digital learning environments, participating in online discussions, and utilizing online learning resources, thereby demonstrating higher levels of online student engagement (Li et al., 2023). Conversely, inadequate IL may increase cognitive burden and learning frustration, ultimately reducing students' willingness and ability to engage in online learning activities. Although previous studies have separately examined the associations among self-directed learning ability, IL, and online student engagement, research investigating the underlying mechanisms linking these variables remains limited, particularly in nursing students (Chang et al., 2021). Most existing studies have focused on general university populations or non-health-related disciplines, while evidence derived from nursing education contexts remains insufficient. Given the unique academic demands of nursing education, including intensive theoretical learning, evidence-based practice, and rapid information updating, understanding the potential mediating role of IL may provide important insights into how self-directed learning ability influences online student engagement among nursing students.

### 1.3 The moderating role of self-control

Self-control refers to an individual's capacity to regulate thoughts, emotions, attention, and behaviors in the presence of impulses, distractions, or competing immediate rewards in order to achieve long-term goals (Gillebaart, 2018). In educational settings, self-control is considered a critical self-regulatory resource that enables students to maintain sustained attention, manage learning behaviors, and resist distractions during the learning process (Gupta et al., 2024). This ability may be particularly important in online learning environments, where external supervision is relatively limited and students are required to independently regulate their learning activities (Zhu et al., 2022). Compared with traditional face-to-face learning, online learning environments expose students to substantially more distractions and temptations, such as social media, online entertainment, and short-form video platforms (Sun et al., 2023). These competing stimuli may interfere with concentration, reduce task persistence, and weaken learning engagement. Under such conditions, students with higher levels of self-control may be better able to suppress impulsive behaviors, maintain attention on academic tasks, and adhere to planned learning activities. Consequently, they may be more capable of effectively translating self-directed learning strategies into actual learning behaviors and sustained engagement. The potential moderating role of self-control can also be understood from the perspectives of self-regulated learning theory and conservation of resources theory (Mazandarani, 2024; Schiff et al., 2025). Meanwhile, conservation of resources theory suggests that individuals rely on internal psychological resources to cope with environmental demands and maintain adaptive functioning (She et al., 2021). Self-control, as an important self-regulatory resource, may help students better cope with the cognitive load and environmental distractions commonly encountered in online learning contexts (Nigg, 2017). In other words, even when students possess strong self-directed learning ability, insufficient self-control may limit their ability to consistently implement effective learning strategies in practice. Previous studies have shown that higher self-control is associated with better academic performance, greater persistence, and more adaptive learning behaviors in digital learning environments (Wang et al., 2022). However, limited research has examined whether self-control influences the strength of the association between self-directed learning ability and online student engagement, particularly among nursing students. Given the high autonomy and self-management demands of nursing education, self-control may play a particularly important role in determining whether students can effectively maintain engagement in online learning environments.

### 1.4 The present study

**Figure 1:**
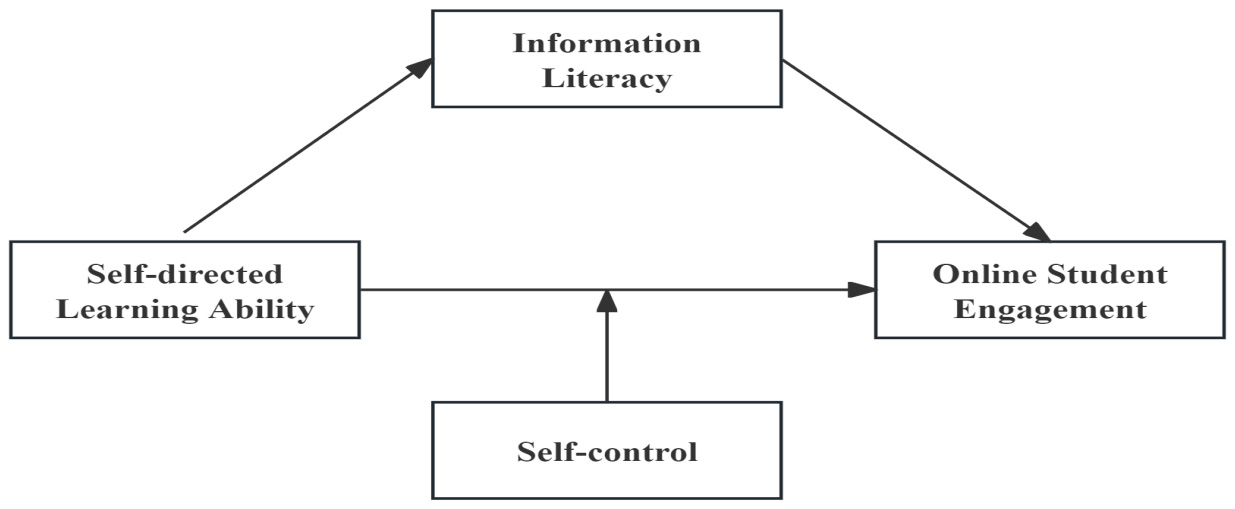
Hypothetical theory model.

A moderated mediation model was developed to examine the relationships and mechanisms linking self-directed learning ability, information literacy, self-control, and online student engagement among nursing students, aiming to provide new insights and theoretical foundations for enhancing the online learning outcomes of nursing students. Hence, as shown in the conceptual model (Figure 1), this study posited the following hypotheses:

H1: Self-directed learning ability significantly predicts online student engagement among nursing students.
H2: Information literacy mediates the association between self-directed learning ability and online student engagement among nursing students.
H3: Self-control moderates the relationship between self-directed learning ability and online student engagement among nursing students.

## 2 Methods

### 2.1 Study design

This cross-sectional study was conducted from September 2025 to December 2025. A convenience sampling approach was employed to recruit undergraduate nursing students. Data were collected through an online survey administered via the Wenjuanxing platform. A total of 1,123 questionnaires were initially collected, from which 917 valid responses were retained after excluding incomplete or invalid submissions, resulting in an effective response rate of 81.6%.

### 2.2 Participants and setting

The study population consisted of undergraduate nursing students enrolled in universities offering nursing programs across five provinces/regions in China: Ningxia, Jilin, Harbin, Kunming, and Dali. Eligibility criteria for participation included: (1) current enrollment as a full-time nursing student; (2) prior participation in at least one semester of online learning courses; and (3) provision of informed consent. Students on leave of absence or those who had not engaged with the online learning platform during the semester were excluded.

### 2.3 Data collection

Data collection was conducted between September 2025 and December 2025. An online survey was developed using the Wenjuanxing platform, a prominent Chinese online survey tool akin to Qualtrics. The survey link was disseminated through class WeChat groups and university learning management systems (LMS). To ensure data quality, the survey incorporated attention checks and a minimum completion time threshold. The study protocol received ethical approval from the Institutional Review Board (IRB) of Ningxia Medical University, and all procedures adhered to the ethical standards outlined in the Declaration of Helsinki.

### 2.4 Measurements

#### 2.4.1 Demographic characteristics??

Participants provided information on their gender, age, year of study, university affiliation, and parental educational and occupational backgrounds. Variables including gender, age, year of study, university affiliation, and parental educational and occupational backgrounds were treated as covariates in subsequent analyses. All covariates were included in all mediation and moderation models.

#### 2.4.2 Key variables

**(1) Online student engagement**

Online student engagement was assessed using the Online Student Engagement (OSE) scale, a 17-item instrument revised by Li and Huang (2010). This scale comprises three dimensions: Motivation (6 items), Vitality/Energy (6 items), and Attention/Concentration (5 items). A representative item is: “I gain a sense of accomplishment from learning nursing knowledge online.” Items are rated on a 5-point Likert scale, ranging from 1 (Strongly disagree) to 5 (Strongly agree). Total scores range from 17 to 85, with higher scores indicating greater levels of engagement. The scale has demonstrated robust reliability and validity within Chinese nursing student populations. In this study, *Cronbach's* α was 0.92.

**(2) Information literacy**

Information literacy was assessed using the 6-Item Information Literacy Scale (6ILS), validated by Kuglitsch and Watkins (2018). This scale comprises a single dimension assessing information literacy, including information retrieval, evaluation, processing, and application. A representative item is: “I can evaluate the quality of the information I find.” Items are rated on a 5-point Likert scale, ranging from 1 (Strongly disagree) to 5 (Strongly agree). Total scores range from 6 to 30, with higher scores indicating greater levels of information literacy. This scale has been validated in Chinese populations and validity compared with clinical evaluation (Zhao, 2024). In this study, *Cronbach's* α was 0.89.

**(3) Self-control**

Self-control was measured using the Brief Self-Control Scale (BSCS), a 7-item instrument revised by Tan and Guo (2008) from the original scale developed by Tangney et al. (2004). This scale assesses the ability to override impulses. An example item is: “I am good at resisting temptation.” Items are scored on a 7-point Likert scale, ranging from 1 (Strongly disagree) to 7 (Strongly agree), with reverse scoring applied to specific items as necessary. Higher scores denote stronger self-control. Relevant validation tests have been carried out in Chinese populations, confirming sound reliability and validity of the scale. The *Cronbach's* α for this study was 0.86.

**(4) Self-directed learning ability**

Self-directed learning ability was assessed using the Self-Directed Learning Readiness Scale (SDLRS), developed by Huang et al. (2007). This 55-item scale evaluates students' attitudes and capacities for engaging in self-directed learning. It encompasses six dimensions: Active Learning, Negative Learning (reverse scored), Effective Learning, Love of Learning, Open Learning, and Lifelong Learning. A representative item for Active Learning is: “I take the initiative to find learning resources to solve problems I don't understand.” Items are rated on a 5-point Likert scale. The SDLRS has been extensively employed in Chinese nursing education research. The *Cronbach's* α for the present study was 0.94.

### 2.5 Ethical considerations

Participation in this study was entirely voluntary and anonymous. Electronic informed consent was obtained from all participants at the commencement of the online survey. Participants were explicitly informed of their right to withdraw from the study at any point without incurring any negative consequences. All collected data were securely stored on password-protected servers, with access strictly limited to the research team.

### 2.6 Statistical analysis

Data were analyzed using SPSS version 26.0 and the PROCESS macro (version 4.2) for SPSS. Means, standard deviations, and frequencies were computed for all study variables. Confirmatory factor analysis (CFA) was conducted using AMOS 24.0 to examine the construct validity of the three-dimensional Online Student Engagement (OSE) scale and the six-dimensional Self-Directed Learning Readiness Scale (SDLRS). Model fit was evaluated using χ^2^/*df* , CFI, TLI, IFI, and RMSEA. Harman's single-factor test was performed to assess for common method variance. Pearson correlation coefficients were calculated to examine the bivariate relationships among self-directed learning (SDL), information literacy, self-control, and online engagement. Model 4 of the PROCESS macro was utilized to investigate the mediating effect of information literacy on the relationship between SDL and online engagement. Bias-corrected bootstrap confidence intervals (5,000 resamples) were employed to determine the significance of the indirect effect. Model 59 was applied to test the moderating role of self-control. Specifically, interaction terms (SDL × Self-Control) were incorporated into the regression equations predicting information literacy and online engagement. Significant interactions were further interpreted through simple slope analyses. Statistical significance was established at a two-tailed *p-value* of < 0.05.

## 3 Results

### 3.1 Participant characteristics and common method bias

A total of 1,123 questionnaires were initially collected. If the missing proportion of items in a single questionnaire is no less than 20%, the questionnaire is deemed to have substantial missing data. Questionnaires with highly identical responses to all items, obvious regular answering patterns, or an actual completion duration shorter than 10 min will be excluded from statistical analysis if any of the above conditions is satisfied. After data screening, 917 valid questionnaires were retained, yielding an effective response rate of 81.6%. Detailed demographic characteristics are presented in Table 1. The mean age of participants was 19.4 years (SD = 1.8). The majority of the sample was female (83%) and comprised students in their second or third year of study.

**Table 1:** Demographic information (*N* = 917).

Variables	*N* (%) or M ±SD
Age	19.4 ± 1.8
Gender	
Man	156 (17.0)
Woman	761 (83.0)
Educational level	
Undergraduate degree students	516 (56.3)
Associate degree students	401 (43.7)
Grade	
Freshman year	330(36.0)
Sophomore year	397 (43.3)
Junior year	156 (17.0)
Senior year	34 (3.7)
Mother's educational attainment	
Primary school and below	335 (36.5)
Junior middle school	333 (36.3)
Senior high school or vocational school	143 (15.6)
Post-secondary (College) and above	106 (11.6)
Father's educational attainment	
Primary school and below	260 (28.4)
Junior middle school	375 (40.9)
Senior high school or vocational school	189 (20.6)
Post-secondary (College) and above	93 (10.1)

Harman's single-factor test revealed the extraction of 14 factors with eigenvalues greater than 1. The first factor accounted for 31.45% of the total variance, which is below the commonly accepted 40% threshold. Consequently, common method bias was not considered a significant concern in this study.

### 3.2 CFA results

The results of confirmatory factor analysis indicated that for the self-directed learning ability scale, χ^2^/*df* = 5.51, CFI = 0.828, TLI = 0.815, IFI = 0.828, and RMSEA = 0.070. For the online student engagement scale, χ^2^/*df* = 6.33, CFI = 0.945, TLI = 0.936, IFI = 0.945, and RMSEA = 0.076. According to Brown's evaluation criteria for CFA fitting indices (Brown, 2015), the CFI, TLI, and IFI values of both scales were higher than 0.80, and RMSEA values were lower than 0.08, reaching an acceptable model fitting level. Although the χ^2^/*df* values were relatively high, this indicator is highly sensitive to sample size. Moreover, McNeish and Wolf (2023) proposed that the traditional fixed threshold of fitting indices lacks universal applicability, as it varies with model characteristics including the number of factors, items and sample size. The goodness-of-fit should be evaluated dynamically based on specific model conditions. Overall, the construct validity of the two scales was acceptable. Full fit indices and confirmatory factor analysis results for both scales are detailed in Table 2.

**Table 2:** Results of confirmatory factor analysis.

Variables	χ^2^/*df*	CFI	TLI	IFI	RMSEA
Self-directed learning ability	5.51	0.828	0.815	0.828	0.070
Online student engagement	6.33	0.945	0.936	0.945	0.076

χ^2^/df, Chi-squared divided by degrees of freedom; CFI, comparative fit index; TLI, tucker-lewis index; IFI, incremental fit index; RMSEA, root mean square error of approximation.

### 3.3 Descriptive statistics and correlations

Table 3 presents the means, standard deviations, and inter-correlations of the study variables. The mean score for online student engagement was 61.15 (SD = 11.79). Self-directed learning ability (M = 165.99, SD = 33.01), information literacy (M = 19.70, SD = 4.12), and self-control (M = 71.83, SD = 13.09) were all significantly and positively correlated with online engagement (*r* = 0.508, 0.715, and 0.685, respectively, all *p* < 0.001). Furthermore, significant correlations were observed among the independent variables, providing a robust foundation for subsequent mediation and moderation analyses.

**Table 3:** Descriptive statistics and correlation analysis (*N* = 917).

Variables	*M*	*SD*	1	2	3	4
1 Online learning engagement	61.15	11.79	1			
2 Self-directed learning ability	165.99	33.01	0.508^**^	1		
3 Self-control	71.83	13.09	0.685^**^	0.511^**^	1	
4 Information literacy	19.70	4.12	0.715^**^	0.552^**^	0.592^**^	1

^**^p < 0.001.

### 3.4 Mediation effect of information literacy

The mediation analysis results are displayed in Table 4. Self-directed learning ability showed a significant positive total effect on online learning engagement (β = 0.511, *p* < 0.001) in Model 1. In Model 2, self-directed learning ability significantly and positively predicted information literacy (β = 0.552, *p* < 0.001). In Model 3, when both self-directed learning ability and information literacy were entered into the regression model, information literacy remained a significant positive predictor of online learning engagement (β = 0.626, *p* < 0.001), whereas the direct effect of self-directed learning ability was reduced but still statistically significant (β = 0.165, *p* < 0.001). Bootstrap analysis confirmed that the indirect effect of self-directed learning ability on online learning engagement via information literacy was 0.346 [95% CI (0.278, 0.418)], accounting for approximately 67.7% of the total effect.

**Table 4:** Mediation effect analysis of the influence of self-directed learning ability on online learning engagement through information literacy (*N* = 917).

Variables	Model 1 (Online learning engagement)				Model 2 (Information literacy)				Model 3 (Online learning engagement)			
	β	*SE*	*t*	95%*CI*	β	*SE*	*t*	95%*CI*	β	*SE*	*t*	95%*CI*
Self-directed learning ability	0.511	0.010	17.586^**^	[0.162, 0.203]	0.552	0.004	19.561^**^	[0.062, 0.076]	0.165	0.010	6.002^**^	[0.040, 0.078]
Information literacy									0.626	0.078	23.071^**^	[1.639, 1.944]
*R^2^*	0.268				0.309				0.529			
*F*	36.848^**^				44.933^**^				105.836^**^			

^**^p < 0.001.

### 3.5 Moderating effect of self-control

Table 5 presents the results of the moderation analysis. Model 4 examined the moderation of the first stage (SDL → Information Literacy). The interaction term (SDL × Self-Control) was not statistically significant (β = −0.121, *p* > 0.05). Model 5 assessed the moderation of the direct path (SDL → Engagement) and the second stage. The interaction term (SDL × Self-Control) was found to be significant and negative (β = −0.310, *p* < 0.01). Conversely, the interaction term for Information Literacy × Self-Control was not significant.

**Table 5:** Moderating effects model analysis of self-control in the pathway from self-directed learning ability to online learning engagement (*N* = 917).

Variables	Model 4 (Information literacy)				Model 5 (Online learning engagement)			
	β	*SE*	*t*	95% *CI*	β	*SE*	*t*	95% *CI*
Self-directed learning ability	0.397	0.009	5.682^**^	[0.032, 0.067]	0.225	0.030	2.670^**^	[0.021, 0.140]
Self-control	0.498	0.042	6.143^**^	[0.176, 0.341]	0.571	0.107	7.946^**^	[0.637, 1.056]
Self-directed learning ability × Self-control	−0.121	0.000	−0.983	[−0.001, 0.000]	−0.310	0.001	−2.140^*^	[−0.003, 0.000]
Information literacy					0.455	0.257	5.063^**^	[0.797, 1.806]
Information literacy × Self-control					−0.002	0.007	−0.016	[−0.014, 0.013]
*R^2^*	0.441				0.628			
*F*	64.800^**^				116.995^**^			

^**^p < 0.001, ^*^p < 0.01.

**Figure 2:**
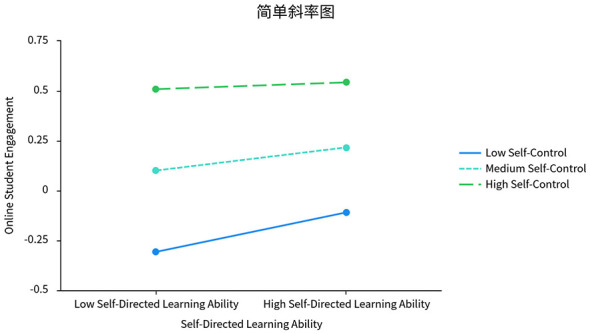
Simple slope plot for the moderating effect.

**Figure 3:**
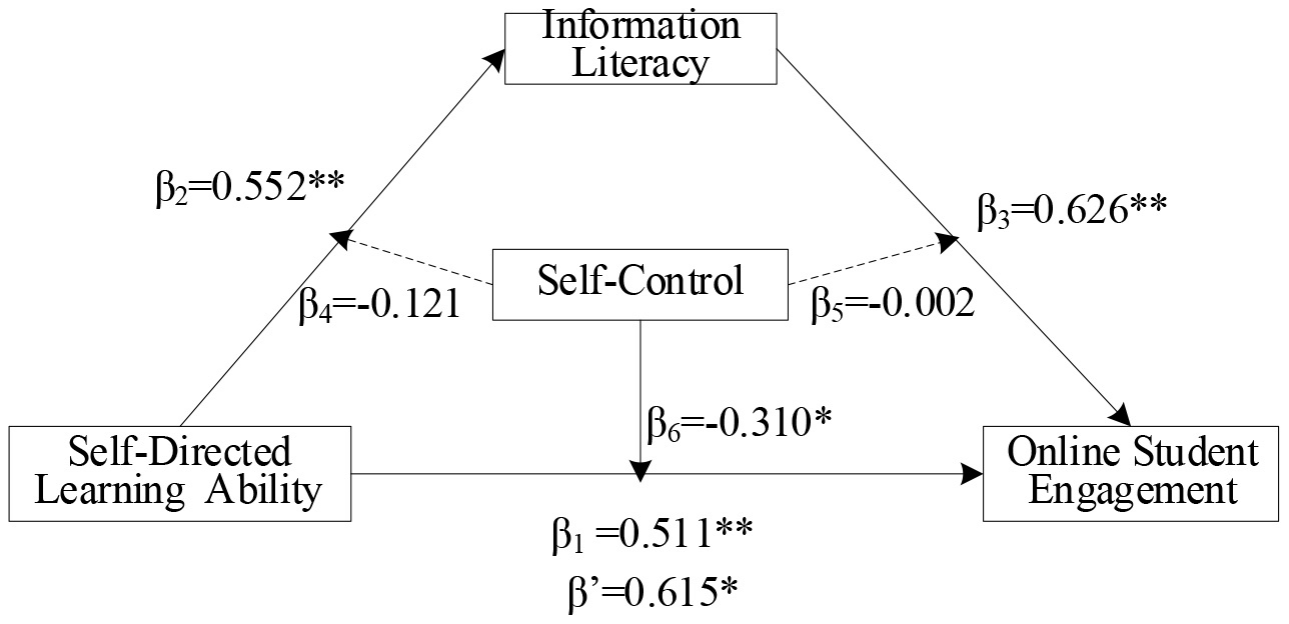
Regulatory mediating model. ^**^p < 0.001, ^*^p < 0.01.

To elucidate the significant interaction, simple slope analyses were conducted (refer to Figure 2). The results demonstrated that for students with lower self-control (1 standard deviation below the mean), SDL exhibited a strong positive predictive effect on engagement (simple slope = 0.65, *p* < 0.001). In contrast, for students with higher self-control (1 standard deviation above the mean), the effect of SDL on engagement was weaker but still positive (simple slope = 0.35, *p* < 0.001). This pattern confirms Hypothesis H3: self-control acts as a buffer, such that the influence of SDL on engagement is less critical when self-control is high, but becomes crucial when self-control is low. Figure 3 depicts the full regulatory mediating model of this study.

## 4 Discussion

This study examined the relationship between self-directed learning ability and online student engagement among nursing students, and further explored the roles of information literacy and self-control in this association. The results showed that self-directed learning ability was positively associated with online student engagement. In addition, information literacy played a mediating role in this relationship, indicating that self-directed learning ability can enhance online student engagement by improving individuals' abilities to access, evaluate, and use information. Meanwhile, self-control served as a moderator in this process, such that the positive effect of self-directed learning ability on online student engagement was stronger among nursing students with lower levels of self-control.

This study found that self-directed learning ability significantly predicted online student engagement among nursing students, supporting H1. With higher levels of self-directed learning ability, online student engagement was also higher, consistent with previous studies (Berre et al., 2025). The present findings may be explained from the perspectives of Student Engagement Theory. Learners are more likely to actively engage in learning activities when their needs for autonomy and competence are supported (Neufeld, 2026; Jones et al., 2026). Self-directed learning ability reflects students' capacity to independently plan, regulate, and manage their own learning processes, which may strengthen their sense of autonomy and perceived learning competence. In online learning contexts, where external supervision and structured guidance are relatively limited, students rely more heavily on intrinsic motivation and self-management abilities to maintain effective learning behaviors (Ryan and Deci, 2000). Students with higher self-directed learning ability show more active participation and sustained involvement in learning tasks (Edisherashvili et al., 2021). Therefore, self-directed learning ability plays an important role in the development of online student engagement. Overall, in educational practice, strengthening training on goal setting and learning management may help improve online student engagement.

This study found that information literacy mediated the relationship between self-directed learning ability and online student engagement among nursing students, supporting H2. Nursing students with stronger self-directed learning ability exhibited higher information literacy and greater online student engagement. These findings align with previous research (Cadorin et al., 2017; Huang et al., 2023). Studies in college students have linked higher information literacy to elevated behavioral, cognitive, and emotional online engagement (Zhao, 2024). While research in medicine students has associated digital health literacy—an essential dimension of information literacy—with increased online student engagement (Rueda-Medina et al., 2023). The present findings may also be explained from the perspective of self-regulated learning theory. This theory emphasizes that learning is an active process involving goal setting, strategy use, self-monitoring, and adjustment (Tinajero et al., 2024). Self-directed learning ability reflects learners' capacity to independently plan, manage, and evaluate their learning activities. In online learning environments, nursing students with stronger self-directed learning ability may be more likely to actively search for learning resources, evaluate information quality, and select appropriate learning strategies. These processes are closely related to information literacy. Information literacy is not only a technical skill for information searching, but also a cognitive and metacognitive ability involving information access, appraisal, integration, and application (Trixa and Kaspar, 2024). It helps students screen reliable resources, relieve information overload and engage efficiently in online learning, acting as a key cognitive link between self-directed learning and online engagement. Strong self-directed learning paired with competent information skills also allows students to adapt well to online learning and keep motivated, focused and interactive. For nursing education, it is advisable to embed information literacy training into curricula to develop students' abilities in information retrieval, screening, assessment and evidence evaluation. In addition, teaching practices focusing on self-directed learning, including independent assignments, reflective exercises, and guided resource exploration, can effectively boost nursing students' online engagement.

This study found that self-control moderates the relationship between self-directed learning ability and online student engagement among nursing students, supporting H3. Online learning is highly autonomous and has limited external constraints (Trixa and Kaspar, 2024). Therefore, students need stronger behavioral regulation to maintain continuous engagement. It also includes many distractions, such as social media and entertainment content (Cockerham et al., 2021), which may interfere with sustained attention during learning. Learning engagement depends on both learning ability and behavioral execution (Wang et al., 2024). This suggests that learning ability alone may not directly translate into actual engagement without effective behavioral regulation. Self-directed learning involves goal setting, strategy selection, and process management. However, its translation into actual engagement requires self-regulation (Tinajero et al., 2024), especially in online learning environments with fewer external controls. Self-regulation theory highlights continuous self-monitoring and interference inhibition (Lengetti et al., 2020). Self-control is a core resource in this process. Nursing students with high self-control can inhibit immediate responses to situational cues. They maintain attention on learning tasks and sustain strategy implementation. This reflects a stable transformation from learning ability to learning behavior. In contrast, students with low self-control show attention shifts and behavioral interruption under external interference. Self-directed learning remains at the level of cognition or intention. Stable engagement is limited. This pattern indicates that self-control weakens the positive relationship between self-directed learning ability and online student engagement.

## 5 Limitations

This study has several limitations. First, the cross-sectional design limits the ability to determine temporal relationships among self-directed learning ability, information literacy, self-control, and online student engagement. Therefore, the observed associations should be interpreted with caution, and causal relationships cannot be established. Future research could use longitudinal or experimental designs to examine the stability and direction of these relationships over time. Second, the study relied mainly on self-reported measures. Such measures may be influenced by social desirability and recall bias, which could affect the accuracy of participants' responses and potentially overestimate or underestimate the observed relationships. Future studies may incorporate behavioral indicators, learning analytics, or objective assessments of information literacy to improve measurement validity.

Third, the sample consisted mainly of nursing students, which may limit the generalizability of the findings to students from other disciplines or educational contexts. Differences in academic demands, learning environments, and digital learning experiences may influence online student engagement. Therefore, future studies should consider multi-center sampling and include students from diverse academic backgrounds. Finally, although self-control was examined as a moderator in this study, online student engagement is likely influenced by multiple individual and contextual factors. Variables such as learning motivation, learning environment, technological support, and instructional design may also play important roles. Future research could explore more comprehensive models to better understand the mechanisms underlying online student engagement.

## 6 Conclusion

This study examined the relationship between self-directed learning ability and online student engagement among nursing students. The mediating role of information literacy and the moderating role of self-control were analyzed. The results showed that self-directed learning ability was significantly associated with online student engagement. Information literacy showed a mediating role. Self-control showed a moderating role in the relationship between self-directed learning ability and online student engagement, with a stronger association at lower levels of self-control. These findings provide empirical support for self-determination theory and conservation of resources theory. They also highlight key psychological mechanisms related to online student engagement among nursing students and provide important evidence for developing targeted interventions and improving the quality of online teaching in nursing education.

Based on the findings, several strategies can be applied to improve online teaching. Instructional design can include support for self-directed learning, such as flexible learning paths and pacing. Information literacy training can be strengthened, including skills in information search, selection, and evaluation. Time management, task segmentation, and behavior monitoring can be used to enhance self-control and reduce distractions in online environments. Learning analytics and formative assessment can be used to provide ongoing feedback and support student engagement. Overall, interventions should focus on both learning abilities and self-regulatory resources, with the aim of improving online student engagement and supporting the quality of online teaching in nursing education.

## Data Availability

The raw data supporting the conclusions of this article will be made available by the authors, without undue reservation.
